# Case-control association testing by graphical modeling for the Genetic Analysis Workshop 17 mini-exome sequence data

**DOI:** 10.1186/1753-6561-5-S9-S62

**Published:** 2011-11-29

**Authors:** Haley J Abel, Alun Thomas

**Affiliations:** 1 Division of Statistical Genomics, Washington University School of Medicine, Saint Louis, MO, USA; 2 Division of Genetic Epidemiology, University of Utah, 391 Chipeta Way, Salt Lake City, UT 84105, USA

## Abstract

We generalize recent work on graphical models for linkage disequilibrium to estimate the conditional independence structure between all variables for individuals in the Genetic Analysis Workshop 17 unrelated individuals data set. Using a stepwise approach for computational efficiency and an extension of our previously described methods, we estimate a model that describes the relationships between the disease trait, all quantitative variables, all covariates, ethnic origin, and the loci most strongly associated with these variables. We performed our analysis for the first 50 replicate data sets. We found that our approach was able to describe the relationships between the outcomes and covariates and that it could correctly detect associations of disease with several loci and with a reasonable false-positive detection rate.

## Background

As the search for genetic variation underlying complex diseases turns to rare variants of small effect ascertained from genome-wide or exome-wide sequence data, the need for methods of disease-gene association that account for the high degree of nonindependence between densely spaced genetic variants (i.e., linkage disequilibrium [LD]) becomes increasingly critical. Failure to consider LD may result in false-positive associations. We have recently shown [1] that graphical modeling is a useful and accurate approach for estimating the LD model, or joint distribution of alleles across loci, for genome-wide single-nucleotide polymorphism (SNP) array data. The use of a general decomposable graphical model for the LD structure has a number of advantages. One such advantage is the ability to incorporate discrete covariates and outcome variables into the graph, so that the associations between outcomes, covariates, and allele states can be estimated without prior knowledge or specification of a model. Here, we apply such an approach to the Genetic Analysis Workshop 17 (GAW17) mini-exome case-control sequence data in an effort to detect genes associated with disease phenotype while controlling for LD as well as sex, age, and smoking habits. Because allele frequencies and patterns of LD are known to vary between populations, we include each individual’s population of origin as an additional covariate.

## Methods

A graphical model describes the joint distribution of a set of random variables as the product of conditional distributions on smaller subsets of the variables. This factorization has a corresponding Markov (or conditional independence) graph, in which vertices represent variables and edges connect variables that appear together in the same conditional distribution (see Lauritzen and Sheehan [2] for background on graphical models). We apply a graphical modeling approach to the GAW17 data to find SNPs associated with disease phenotype and with quantitative traits Q1, Q2, and Q4 while controlling for LD and several discrete covariates. Specifically, we modified the method detailed by Abel and Thomas [1] to estimate a decomposable graphical model for the joint distribution  using the Markov chain Monte Carlo (MCMC) method. Here *Y* is the disease phenotype; the  are the discretized (into thirds) quantitative traits; the  are the discrete covariates (population of origin, smoking status, sex, and age) (dichotomized by the median); and the  are the alleles at loci 1 through *n*.

For LD models for SNP genotype arrays, the structure of the conditional independence graphs tends to be long and thin, facilitating efficient computation. However, because of, for example, allele frequencies, age structure, and smoking rates that vary across populations, the incorporation of outcome variables and covariates into the model results in a more complex stellate (and thus computationally taxing) structure. We use a stepwise iterative approach to render the problem more computationally tractable.

In the first step of our algorithm we estimate for all nonsynonymous variants on each chromosome the posterior most-probable population-dependent graphical model. That is, we empirically estimate a joint distribution on the ethnic origin variable and alleles at all loci with nonsynonymous variants [3]. (In general, exclusion of synonymous variants is a reasonable simplification, although synonymous mutations could induce a change of phenotype by means of, for instance, codon bias. We note that all GAW17 simulated causal variants were chosen to be nonsynonymous.) These models are estimated using a slight modification of the program FitGMLD, described by Abel and Thomas [1].

Briefly, we start with arbitrarily phased haplotypes and an initial configuration for the graph in which the dependence structure for the loci is a second-order Markov chain and in which all loci are associated with the population of origin. The best-fitting graphical models are then estimated using simulated annealing through a method that iterates between rounds of decomposability-preserving Metropolis graph updates and blocked Gibbs phase updates based on the current structure of allele associations [1,3]. In the Metropolis update of an incumbent graph *G*, a new graph *G*′ is proposed by randomly connecting or disconnecting an appropriate pair of vertices [4]. The proposed graph *G*′ is then accepted with probability:(1)

otherwise the graph *G* remains. Here, *t* is the temperature parameter for the simulated annealing, and the penalized likelihood:(2)

serves as an information criterion in which the penalty depends on the graph’s degrees of freedom (df).  is the maximized graph likelihood, and *α* is a constant. To allow for an efficient walking-windowed estimation, we assume that alleles separated by more than 15 intervening loci are conditionally independent. We then use the obtained model to estimate the most probable phase of each genotype. We note that, although necessary in general, this step may not be required for the previously phased GAW17 data; however, it would help to mitigate any phasing or genotyping errors.

The second and third steps of the algorithm are also carried out for each chromosome individually. In these steps, the haplotype phase is fixed according to the results of the first stage, and model fitting proceeds using Metropolis graph updates only. In the second step, a restricted graphical model is fitted to the full data set, including all loci, disease status, quantitative traits, and covariates. To reduce the model space and improve mixing, we enforce the restriction that edges can connect loci separated by at most 50 SNPs. We then prune the Markov graph to include only a subset of loci that are most strongly associated with any of the outcomes or covariates; that is, we restrict the subgraph to spanning all neighbors of all outcomes and covariates, with the exception of ethnic origin, up to third degree. At this stage we exclude the loci linked only to ethnic origin because they are unlikely to be relevant predictors of disease.

The third step is similar to the second; at this stage, however, because of the decreased model space, we fit an unrestricted graphical model to the reduced data set. We then perform a second pruning step, this time restricting the new Markov graph to the subgraph of all first- and second-degree neighbors of all loci except ethnic origin.

In the final stage of our algorithm, we combine the remaining loci from all chromosomes to determine the loci that are most strongly associated with any of the outcomes while controlling for ethic origin and all covariates. Again, we fit an unrestricted graphical model to the data. The nearest neighbors of the disease state or any of the quantitative traits are considered significantly associated (according to the information criterion given by Eq. (2)).

## Results

**Figure 1:**
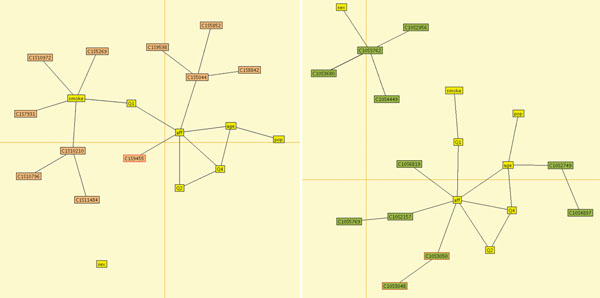
**Typical pruned Markov graphs after the third model-fitting stage of our algorithm**. Outcome variables and covariates are shown in yellow. Correctly identified variants are indicated by red circles. Left panel: chromosome 1. Right panel: chromosome 10.

**Figure 2:**
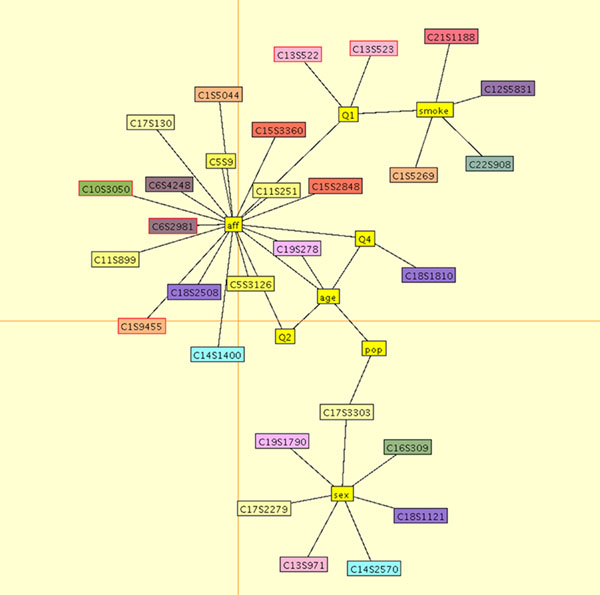
**Markov graph estimated in the final model-fitting step.** SNPs are color-coded by chromosome. Outcome variables and covariates are shown in yellow. Correctly identified genes are indicated by red circles.

We performed our analysis for the first 50 replicates of the GAW17 data set. In all cases, the estimated relationships between disease status, the quantitative traits, and the measured covariates closely approximate those specified in the model used to generate the data. To illustrate the method, we depict in Figures 1 and 2 the intermediate and final model estimates for the first replicate. Typical results for two chromosomes after the third model-fitting stage are shown in Figure 1. In this instance, on chromosome 1 we detect one causal gene and one noncausal haplotype (consisting of four nonindependent loci) associated with disease status. This model suggests a direct association of trait Q1 with disease and more complex interactions between age, Q2, and disease and between Q2, Q4, and disease. Our model for chromosome 10 detects one causal haplotype and one noncausal haplotype and a single locus associated with disease status. The pattern of associations between covariates and outcomes is similar. Although our main interest here is to detect associations between variables, our program estimates marginal and conditional distributions on all subsets of variables, indicating, for instance, that the probability of disease decreases with increasing quantitative trait Q4.

The final estimated graphical model for the first replicate is shown in Figure 2. For this data set, our method detected five causal variants on four genes. Our model found three variants associated with disease status: C1S9445 in *PIK3C2B*, C6S2981 in *VEGFA*, and C10S3050 in *SIRT1*. All three of these variants are rare, with minor allele frequencies (MAF) of 0.0029, 0.0022, and 0.0022, respectively, and they were simulated to have additive mean effects *β* = 0.23, *β* = 1.2, and *β* = 0.97, respectively. We also detected two common variants, both in *FLT1*, associated with quantitative trait Q1: C13S522 (MAF = 0.028, *β* = 0.62) and C13S523 (MAF = 0.067, *β* = 0.65). Our algorithm also returned 13 noncausal variants (i.e., variants that were not part of the disease model). We note that, as seen in Figure 2, our best-fitting graphical models sometimes connect SNPs that influence traits Q1 and Q2 to disease status (rather than the vertices representing traits Q1 and Q2); however, this confounding is not unexpected or problematic because the disease liability was simulated as a linear combination of latent liability and the three quantitative traits (overall liability = latent liability + Q1 + Q2 − Q4).

Over the first 50 simulated data sets, our graphical modeling method identified, on average, 1.4 SNPs in causal genes per data set. Of these correctly identified SNPs, 90.2% were exactly the causal SNPs rather than SNPs located elsewhere in the causal gene. Our algorithm also identified an average of 15.8 noncausal variants per replicate. Table 1 summarizes our results for the 50 replicates. SNPs are categorized as rare (MAF < 0.005) or common and as having weak (*β* < 0.5) or strong effect; the number of times (out of 50) that each variant was detected is indicated. Out of seven possible variants in the common and strong category, our algorithm detected three: C13S523 (*FLT1*) was seen in 25 replicates, C13S522 (*FLT1*) in 23 replicates, and C1S6533 (*ARNT*) in 2 replicates. Of the 57 variants in the strong and rare category, 6 were seen in at least one replicate. Of 17 common and weak variants, we observed only 1. Finally, out of 81 variants in the rare and weak category, only 1 was detected but in four distinct data replicates.

**Table 1:** Detected causal variants categorized by minor allele frequency and strength of effect

Minor allele frequency	*β* < 0.5	*β* < 0.5
<0.005	C10S3110, *SIRT1* (4)	C1S9445, *PIK3C2B* (1) C3S4873, *BCHE* (2) C6S2981, *VEGFA* (1) C9S376, *VLDLR* (1) C10S3048, *SIRT1* (1) C10S3050, *SIRT1* (1)
≥0.005	C18S2492, *PIK3C3* (1)	C1S6533, *ARNT* (2) C13S522, *FLT1* (23) C13S523, *FLT1* (25)

Causal variants detected out of 50 replicates. Numbers in parentheses denote the number of times (replicates) a particular variant was found. For reference, there were 7 simulated variants in the common and strong category, 57 in the rare and strong category, 81 in the rare and weak category, and 17 in the common and weak category.

## Discussion and conclusions

We have shown that a graphical modeling approach can detect loci associated with complex diseases. Our method also provides a good approximation of the true associations between outcomes and covariates specified by the model. Furthermore, our method has the ability to detect environmental effects on disease as well as gene-gene and gene-environment interactions. We have demonstrated that our method has reasonable power to detect common variants of strong effect, finding mutations in the *FLT1* gene in more than 70% of replicates. Furthermore, our approach shows some power to detect rare variants of large effect, detecting 6 of 57 such variants in at least one replicate. In its current implementation, our method has little power to detect variants with weak effect on phenotype; however, it did find the C10S3110 mutation in *SIRT1* (MAF = 0.002152, *β* = 0.10) in four distinct replicates, probably because of LD between this locus and other rare mutations of stronger effect within the same gene. In general, however, our graphical modeling method is successful at controlling for LD, because it is able to pinpoint causal SNPs within causal genes more than 90% of the time.

Our graphical modeling approach, which controls for population structure, linkage disequilibrium, and relevant covariates, shows considerable promise for detection of both common and rare variants from exome sequence data. However, it does have some limitations that should be addressed in future work. First, our method is purely statistical and avoids biological assumptions (except in its exclusion of synonymous variants); hence it has only weak power to detect rare variants and also tends to detect associated but noncausal variants. Inclusion of, in particular, biological pathway information into the model would help to increase power and decrease detection of spurious associated SNPs. Second, our algorithm uses simulated annealing to find the single most likely graph to represent each data set; using the MCMC method to average over many models from the posterior distribution might help to decrease the number of false positives. By identifying only the nearest neighbors of the outcome variables in the most likely model, our algorithm sometimes excludes causal SNPs in favor of other tightly linked causal or noncausal SNPs. Model averaging and incorporation of, for instance, pathway information into the model would help to increase both sensitivity and specificity by appropriately constraining the model space and considering a larger number of possible solutions. Finally, our graphical models incorporate a single (given) ethnicity variable. In general, this could pose a problem because of admixture or possibly unreliable self-reported ethnicity. We are currently developing a method to extend our LD model (the empirically estimated joint distribution of genotypes) to allow the ethnicity variable to change between loci and small clusters of loci, thus allowing for admixed and unknown ethnic origins.

Despite these limitations, the flexible method of graphical modeling provides a promising approach to the challenge of detecting the genetic variation that underlies complex disease. Given such a disease that involves environmental factors and many rare variants of small effect, inclusion of relevant biological information into the model would help to elucidate the true underlying factors. Graphical modeling might provide a framework for such a systems biological tack.

## Competing interests

The authors declare that there are no competing interests.

## Authors’ contributions

HA performed the analysis and wrote the manuscript. Both authors created the software and read and approved the manuscript.
